# High Expression of Antiviral and Vitamin D Pathway Genes Are a Natural Characteristic of a Small Cohort of HIV-1-Exposed Seronegative Individuals

**DOI:** 10.3389/fimmu.2017.00136

**Published:** 2017-02-13

**Authors:** Wbeimar Aguilar-Jimenez, Irma Saulle, Daria Trabattoni, Francesca Vichi, Sergio Lo Caputo, Francesco Mazzotta, Maria T. Rugeles, Mario Clerici, Mara Biasin

**Affiliations:** ^1^Grupo Inmunovirología, Facultad de Medicina, Universidad de Antioquia UdeA, Medellín, Colombia; ^2^Dipartimento di Scienze Biomediche e Cliniche-Luigi Sacco, Università Degli Studi di Milano, Milan, Italy; ^3^Ospedale S. Maria Annunziata, Florence, Italy; ^4^Dipartimento di Fisiopatologia Medico-Chirurgica e dei Trapianti, Università degli Studi di Milano, Milan, Italy; ^5^Fondazione Don C. Gnocchi, IRCCS, Milan, Italy

**Keywords:** HIV-1, HIV-1-exposed seronegative individuals, vitamin D, antiviral agents, natural resistance

## Abstract

Natural resistance to HIV-1 infection is influenced by genetics, viral-exposure, and endogenous immunomodulators such as vitamin D (VitD), being a multifactorial phenomenon that characterizes HIV-1-exposed seronegative individuals (HESNs). We compared mRNA expression of 10 antivirals, 5 immunoregulators, and 3 VitD pathway genes by qRT-PCR in cells of a small cohort of 11 HESNs, 16 healthy-controls (HCs), and 11 seropositives (SPs) at baseline, in response to calcidiol (VitD precursor) and/or aldithriol-2-(AT2)-inactivated HIV-1. In addition, the expression of TIM-3 on T and NK cells of six HCs after calcidiol and calcitriol (active VitD) treatments was evaluated by flow cytometry. Calcidiol increased the mRNA expression of *HAVCR2* (TIM-3; Th1-cells inhibitor) in HCs and HESNs. AT2-HIV-1 increased the mRNA expression of the activating VitD enzyme *CYP27B1*, of the endogenous antiviral proteins *MX2, TRIM22, APOBEC3G*, and of immunoregulators *ERAP2* and *HAVCR2*, but reduced the mRNA expression of VitD receptor (*VDR*) and antiviral peptides *PI3* and *CAMP* in all groups. Remarkably, higher mRNA levels of *VDR, CYP27B1, PI3, CAMP, SLPI*, and of *ERAP2* were found in HESNs compared to HCs either at baseline or after stimuli. Furthermore, calcitriol increases the percentage of CD4+ T cells expressing TIM-3 protein compared to EtOH controls. These results suggest that high mRNA expression of antiviral and VitD pathway genes could be genetically determined in HESNs more than viral-induced at least in peripheral blood mononuclear cells. Moreover, the virus could potentiate bio-activation and use of VitD, maintaining the homeostasis of the immune system. Interestingly, VitD-induced TIM-3 on T cells, a T cell inhibitory and anti-HIV-1 molecule, requires further studies to analyze the functional outcomes during HIV-1 infection.

## Introduction

The hallmark of the resistant phenotype exhibited by HIV-exposed seronegative individuals (HESNs) seems to be a potent but focused and tightly regulated innate antiviral response, clearing the virus while avoiding excessive immune activation and thus the susceptibility of target cells (1–3).

Antiviral factors such as Elafin (encoded by *PI3* gene) (4, 5), secretory leukocyte protease inhibitor (SLPI) (6), defensins (3, 7, 8), cathelicidin (encoded by *CAMP* gene) (8), APOBEC3G (5, 9), and some members of the antiviral ribonuclease A family (RNases) (3, 5) have been demonstrated to play a role in the natural resistance exhibited by HESNs. Furthermore, some reports have shown that T-cell immunoglobulin and mucin domain 3 (TIM-3) (encoded by *HAVCR2* gene), a negative regulator of the activation of Th1 and Th17 cells (10, 11) can reduce HIV-1 infection *in vitro* (12, 13). Yet, a negative role during chronic progressive HIV-1 infection cannot be ruled out since it has been reported TIM-3 may dampers cytotoxicity of CD8+ T cells in chronically HIV-1-infected subjects (14).

Recently, we and other authors have reported that Vitamin D (VitD), a key immunoregulatory element capable of decreasing inflammation while inducing the expression of antimicrobial peptides (15, 16), reduces HIV-1 infection *in vitro* (17–19). This effect is most likely mediated by the induction of an HIV-1-restrictive less proliferative immunophenotype, reducing viral co-receptor expression while promoting the expression of antiviral genes.

Interestingly, higher plasma VitD levels and higher mRNA expression of VitD receptor (VDR) were found in blood and mucosa from Colombian HESNs compared to unexposed healthy controls (HCs) (20). Furthermore, *VDR* mRNA expression was positively correlated with the expression of antiviral molecules in HESNs (8, 20).

The immune cells are endorsed with the metabolic machinery such as the 1α-hydroxilase (CYP27B1) and the VDR, allowing the bio-activation and use of precursor forms of VitD. This in turn promotes the transcription of genes having VitD response elements (VDREs) in their genomic sequences, such as CYP24A1, a well-known VitD-target gene (21, 22). However, whether an effective bio-activation of VitD results in the expression of antiviral molecules in immune cells from HESNs has not been established yet. Furthermore, since the resistant phenotype could be naturally manifested by genetic predetermination or triggered by viral exposure, differences in RNA expression of immunoregulatory, antiviral, and VitD-related genes was explored in peripheral blood mononuclear cells (PBMCs) of HESNs compared to HCs and seropositives (SPs) from an Italian cohort.

## Materials and Methods

### Study Population

Blood samples were collected from 11 Italian HESNs, their HIV-infected partners (SPs), and from 16 HCs enrolled at the Santa Maria Annunziata Hospital in Florence. Inclusion criteria for the HESNs were a history of multiple unprotected sexual episodes for more than 4 years at the time of enrollment, with at least three episodes of at-risk intercourse within 4 months prior to study entry, and an average of 30 (range, 18 to >100) reported unprotected sexual contacts per year with a SP partner. Infection in HESNs and HCs subjects was ruled out by plasma HIV RNA analyses. None of the subjects included in the study were intravenous drug users.

We have been following this cohort of individuals for the past 12 years (it was established in Tuscany in 1997). Both, the HESNs and HCs are involved in monogamous heterosexual relationships, are part of long-lasting couples, and have very similar sexual activities. The HESNs, SPs, and HCs have similar demographic background: age (mean years ± SD: 51.3 ± 9.2 for HESNs; 51.9 ± 9.2 for SPs, and 47.7 ± 12.9 for HCs), gender (36.4, 63.6, and 45.4%, males for HESNs, SPs, and HCs, respectively), and citizenship as well as share the same genetic background (European–Tuscan ascendancy) and the same exposure to environmental factors. The presence of any chronic disease was an exclusion criterion when the HESN and HC were recruited and no other pathologies were detected at sampling in any of the individuals.

In the SP patients, the median (range) of CD4 cell counts were 575 cells/mL (222–1,184 cells/mL), and viral loads were under the detection limit (>20 copies/mL). All the patients were undergoing antiretroviral (ARV) treatment at the time of the study.

In addition, samples of 6 Colombian HCs were also included to analyzed TIM-3 expression by flow cytometry. The study was designed and performed according to the Helsinki declaration (1975 revised in 2000) and was approved by the Ethics Committee of the Hospital S. Maria Annunziata. All subjects provided written informed consent to participate in this study.

### AT2-Inactivated HIV-1 Viral Stimuli Assays

The PBMCs were isolated by centrifugation on a Ficoll discontinuous density gradient (Lympholyte-H, Cederlane Laboratories). After viability assessment, 2 × 10^6^ PBMCs were resuspended in RPMI 1640 medium (Euroclone, Milan, Italy) supplemented with 20% fetal bovine serum (FBS) (Life sciences), and calcidiol [25(OH)D; VitD precursor] (Sigma-Aldrich) at 100 ng/mL (250 nM) [within the physiological range: 32–100 ng/mL (23)] or EtOH 0.01% v/v as vehicle control for 24 h. Subsequently, 1 × 10^6^ cells were stimulated for 24 h with or without 300 ng of Aldrithiol-2 (AT2)-inactivated R5-tropic HIV-1_Ba-L_ p24 equivalents, maintaining their respective calcidiol- or EtOH-supplemented medium. Inactivation of HIV-1 with AT-2 renders the virus reverse transcription deficient while preserving the functional integrity of the envelope (24). The cells were harvested 24 h posttreatment and kept at −80°C in Isol-RNA lysis reagent (5Prime, Hilden, Germany) until RNA extraction.

### Real-time Retro-Transcribed (RT)-PCR to Test Calcidiol Effects on Gene Expression

A total of 18 genes, belonging to the VitD pathway (CYP27B1, CYP24A1, and VDR), antiviral response (*APOBEC3G, SLPI, PI3, TRIM22, RNASE4, ANG, CAMP, CH25H*, and *MX2*), and immunoregulators (*HAVCR2, FOXP3, NFKBIA, ERAP2*, and *TLR2*) as well as the viral co-receptor *CCR5* were selected for gene expression assays for their relevance in the context of HIV-1 infection [reviewed in Ref. (3, 8, 20, 25, 26)].

The RNA was extracted by Isol-RNA lysis reagent (5Prime) from freshly isolated unstimulated PBMCs (baseline condition) as well as from PBMCs treated with calcidiol/EtOH alone or calcidiol/EtOH plus AT2-HIV-1. Additionally, RNA from calcidiol- or EtOH-treated PBMCs of HCs, stimulated with LPS (Lipopolysaccharide) for 12 h (1 µg/mL, *n* = 6 in each group) were also analyzed as positive controls of *CYP27B1* mRNA expression (27, 28). Following DNAse I treatment (Promega, Madison, WI, USA), RNAs were RT using the Moloney murine leukemia virus retro-transcriptase (Promega. Fitchburg, WI, USA), and reverse transcriptase negative controls were performed to rule out contamination with genomic DNA in PCR amplifications. Real-time RT-PCRs were performed using the iTaq™ Universal SYBR Green^®^ Supermix (Bio-Rad) (genes and primers are detailed in Table S1 in Supplementary Material), running melting curves to ensure specific amplification.

The results are presented as the median of the relative expression units (RUs) to the glyceraldehyde-3-phosphate dehydrogenase (GAPDH), and hypoxanthine-guanine phosphoribosyltransferase (HPRT) reference genes calculated by the ΔCt method using the CFX manager 3.1 (Bio-Rad). Samples that did not amplify in the RT-PCR were excluded from the analysis of the respective gene. The number of samples and the median and interquartile range of mRNA expression of each gene in each condition is detailed in Table S1 in Supplementary Material.

### Flow Cytometry to Test Calcidiol and Calcitriol Effects on TIM-3 Expression on T and NK Cells

In 96-well plates (BD, Franklin Lakes, NJ, USA), 100,000 viable PBMCs/well of 6 Colombian HCs were resuspended in 200 µL RPMI 1640 medium (Gibco, Grand Island, NY, USA) supplemented with 10% FBS and were treated with calcidiol at 250 nM and calcitriol (active form of VitD) at 0.5 nM (both within the physiological range) or with 0.01% v/v EtOH as vehicle control.

Cells were harvested 48 h posttreatments in polypropylene tubes, centrifuged at 700 × *g* for 5 min and washed with PBS. Extracellular staining was done with fluorochrome-labeled antibodies purchased from eBioscience (Santa Clara, CA, USA): fixable viability dye eFluor 506, anti-CD3 PE-Cy7, anti-CD4 PE, anti-CD8 eFluor^®^ 450, anti-CD56 PE-Cy7, and anti-TIM-3 APC-Cy7. Samples were acquired on a BD-LSRFortessa™ flow cytometer, and data were analyzed in FACSDiva v.8.0.1 software. The gating strategies are shown in Figure S1 in Supplementary Material.

### Statistical Analysis

Data were analyzed on the GraphPad Prism v.7.00 software. Normality was tested by the Shapiro–Wilk’s test. One-way ANOVA with *post hoc* Bonferroni’s multiple comparisons or Kruskal–Wallis with Dunn’s multiple comparisons tests were used to compare differences in mRNA at baseline conditions between HESNs and HCs or vs. SPs and to compare differences in percentage and mean fluorescence intensity (MFI) of TIM-3 in T and NK cells between each of the VitD treatments and the vehicle control. Two-way ANOVA and *post hoc* Bonferroni’s multiple comparisons were used to compare differences between calcidiol and the vehicle controls as well as between HESNs vs. HCs and HESNs vs. SPs in AT2-HIV-stimulated or -unstimulated cells. Paired *t-* or Wilcoxon-tests were used to compare differences in mRNA levels between AT2-HIV-stimulated and -unstimulated cells. The correlations between *VDR* mRNA and transcript levels of the molecules analyzed were evaluated using the Spearman coefficient rank (*r*). Two-tailed hypotheses were taken into account, and a *p*-Value <0.05 corrected by multiple testing was considered statistically significant. The results are presented as mean with 95% confidence interval or median with interquartile range. The open-access database of transcription factor binding profiles (http://jaspar.genereg.net/cgi-bin/jaspar_db.pl) was used to find putative VDRE sequences in the genes potentially modulated by VitD in gene expression analysis.

## Results

### HESNs Have a Higher Expression of VitD Pathway Molecules, Suggestive of an Active VitD Metabolism

**Figure 1:**
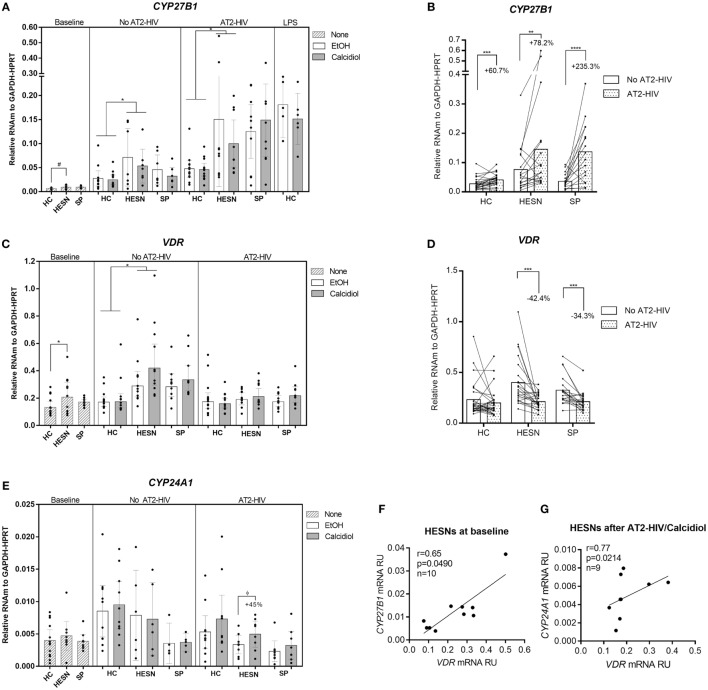
**HIV-exposed seronegative individuals (HESNs) had a higher expression of vitamin D (VitD) pathway molecules, suggestive of an active VitD metabolism**. Peripheral blood mononuclear cells (PBMCs) from HESNs, HCs, or seropositives (SPs) unstimulated (baseline, striped bars) or cultured with calcidiol 100 ng/mL (gray bars) or EtOH 0.01% v/v (white bars) with or without AT2-HIV-1 and mRNA of the VitD pathway genes *CYP27B1* **(A,B)**, VDR **(C,D)**, and *CYP24A1* **(E)** relative to the glyceraldehyde-3-phosphate dehydrogenase/hypoxanthine-guanine phosphoribosyltransferase mRNA levels were quantified by qRT-PCR. LPS-stimulated PBMCs from HCs (1 µg/mL, *n* = 6 in each group) were also analyzed as positive control for *CYP27B1* mRNA expression. Differences between HESNs and HCs or vs. SPs in PBMCs at baseline were analyzed by Kruskal–Wallis for *CYP27B1* mRNA and by one-way ANOVA for *VDR* and *CYP27B1* mRNA. Differences between HESNs and HCs or vs. SPs as well as between calcidiol and EtOH treatments in mRNA expression in PBMCs stimulated with or without AT2-HIV-1 were analyzed by two-way ANOVA. Calcidiol and EtOH treatments were combined to analyze differences in the mRNA levels of *CYP27B1* **(B)** and VDR **(D)** between AT2-HIV-stimulated (dotted bars) and -unstimulated cells (white bars) by paired *t*-test. Correlations between mRNAs of *VDR* and *CYP27B1* **(F)** or *CYP24A1* **(G)** in HESNs at baseline were evaluated using the Spearman coefficient rank (*r*). Bars correspond to means with CI 95%. ^ϕ^45% increase: ^ϕ^*p* = 0.3192, ^#^*p* = 0.0698, *p < 0.05, **p < 0.01 are presented.

We first tested for differences in mRNA expression of VitD pathway molecules *VDR, CYP27B1*, and *CYP24A1* between HESNs vs. HCs or HESNs vs. SPs, which could suggest variations in the ability to metabolize calcidiol into the active form calcitriol. As shown in Figure 1A, *CYP27B1* mRNA levels were higher, although not statistically significant, in HESNs compared to HCs at baseline conditions (means: 0.0094 vs. 0.0068 mRNA RU, respectively; *p* = 0.0698). Nonetheless, *CYP27B1* mRNA levels were significantly higher in HESNs than HCs after 48 h of culture in presence of both calcidiol and EtOH (means: 0.0623 vs. 0.0261 mRNA RU, for HESNs and HCs, respectively; *p* = 0.0131). Moreover, similar to LPS, a known inducer of *CYP27B1* expression (29), AT2-HIV-1 stimuli increased mRNA levels of this enzyme to similar levels (Figures 1A,B); this increase was higher in HESNs than HCs regardless calcidiol or EtOH treatments (means: 0.1251 vs. 0.0467 mRNA RU, for HESNs and HCs, respectively; *p* = 0.0487. Figure 1A). Moreover, the AT2-HIV-induced increase in *CYP27B1* mRNA was higher in previously infected SP individuals (235.3%; *p* < 0.00001), compared to HESNs (78.2%; *p* = 0.0056), and HCs (60.7%; *p* = 0.0003) (Figure 1B).

Similarly, as shown in Figure 1C, higher *VDR* mRNA levels were found in HESNs compared to HCs at baseline (means: 0.2432 vs. 0.1477 mRNA RU, respectively; *p* = 0.0232) and after 48 h of culture, regardless of calcidiol or EtOH treatments (means: 0.3995 vs. 0.2135 mRNA RU, for HESN and HCs, respectively; *p* = 0.0020). Furthermore, 48 h of calcidiol or EtOH increased the *VDR* mRNA levels taking into account all three groups (*p* = 0.0424 by two-way ANOVA), but no differences in *VDR* mRNA expression were observed between calcidiol and EtOH treatments nor between any of the groups studied after AT2-HIV-1 stimuli. However, the viral stimulus reduced mRNA expression of *VDR* in HESNs (42.4%; *p* = 0.0001) and in SPs (34.3%; *p* = 0.0002), but not in HCs (Figures 1C,D).

No differences in *CYP24A1* mRNA expression were found in any of the conditions between the groups studied. Although calcidiol increased by 45% mRNA levels of *CYP24A1* in HESNs after AT2-HIV-1 stimulus, the difference was not significant (*p* = 0.3192; Figure 1E).

In addition, mRNA levels of *VDR* were positively correlated with those of *CYP27B1* at baseline conditions (*r* = 0.65, *p* = 0.0490; Figure 1F) and with those of *CYP24A1* after calcidiol plus AT2-HIV-1 stimuli only in HESNs (*r* = 0.77, *p* = 0.0214; Figure 1G).

### HESNs Have Higher Expression of Antiviral Factors Compared to HCs

**Figure 2:**
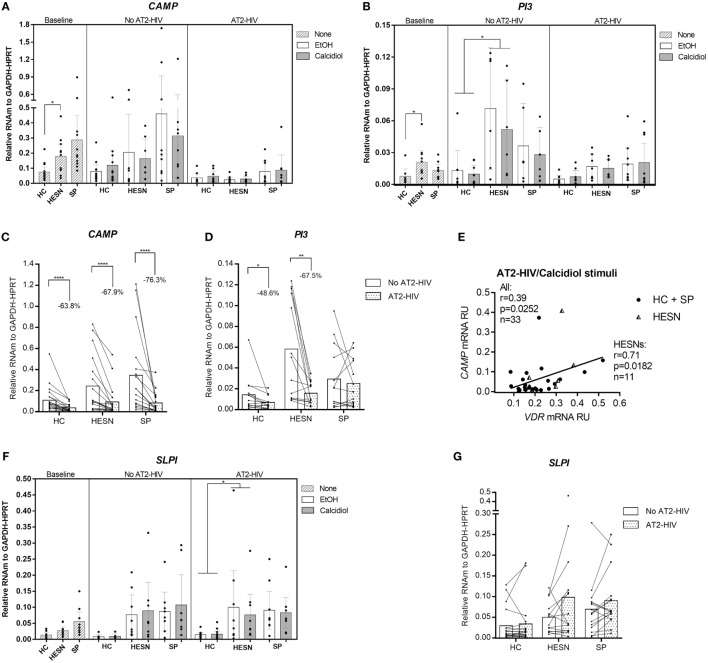
**Higher expression of antiviral genes in HIV-exposed seronegative individuals (HESNs) compared to HCs at baseline conditions, and after viral stimulus**. Peripheral blood mononuclear cells (PBMCs) from HESNs, HCs, or seropositives (SPs) unstimulated (baseline, striped bars) or cultured with calcidiol 100 ng/mL (gray bars) or EtOH 0.01% v/v (white bars) with or without AT2-HIV-1 and mRNA of the antiviral genes *CAMP* (*n* = 7–13) **(A)**, *PI3* (*n* = 6–10) **(B)**, and secretory leukocyte protease inhibitor (SLPI) (*n* = 6–12) **(F)**, relative to the glyceraldehyde-3-phosphate dehydrogenase/hypoxanthine-guanine phosphoribosyltransferase mRNA levels were quantified by qRT-PCR. Differences in mRNA expression between HESNs and HCs or vs. SPs in PBMCs at baseline were analyzed by Kruskal–Wallis. Differences between HESNs and HCs or vs. SPs as well as between calcidiol and EtOH treatments in mRNA expression in PBMCs stimulated with or without AT2-HIV-1 were analyzed by two-way ANOVA. A correlation between mRNAs of *VDR* and *CAMP* **(E)** in PBMCs under AT2-HIV/calcidiol stimuli were evaluated using the Spearman coefficient rank (*r*). Calcidiol and EtOH treatments were combined to analyze differences in the mRNA levels of *CAMP* **(C)**, *PI3* **(D)**, and SLPI (*n* = 6–12) **(G)** between AT2-HIV-stimulated (dotted bars) and -unstimulated cells (white bars) by paired *t*-test. Bars correspond to means with CI 95%. *p < 0.05, **p < 0.01 are presented.

Higher mRNA levels of the *CAMP* were found in HESNs compared to HCs at baseline conditions (medians: 0.1877 vs. 0.0572 mRNA RU, respectively. *p* = 0.0300; Figure 2A). Likewise, higher mRNA levels of the anti-protease *PI3* (encoding elafin) were found in HESNs compared to HCs at baseline conditions (medians: 0.0176 vs. 0.0043 mRNA RU, respectively. *p* = 0.0134; Figure 2B), remaining higher in HESNs than HCs after 48 h culture, regardless of calcidiol or EtOH treatments (means: 0.0611 vs. 0.0115 mRNA RU, for HESN and HCs, respectively, *p* = 0.0165; Figure 2B). There were no significant differences in the mRNA expression of *CAMP* and *PI3* between the studied groups under any of the treatments after AT2-HIV-1 stimulus. Unexpectedly, the viral stimulus reduced mRNA expression of *CAMP* in HCs (63.8%; *p* < 0.0001), in HESNs (67.9%; *p* < 0.0001), and in SPs (76.3%; *p* < 0.0001) (Figure 2C). The viral stimulus also reduced mRNA expression of *PI3* in HCs (48.6%; *p* = 0.0257), in HESNs (67.5%; *p* = 0.0015), but not in SPs (Figure 2D). In addition, after AT2-HIV-1 stimulus, *CAMP* mRNA positively correlate with the VDR mRNA in all individuals (*r* = 0.39, *p* = 0.0252; Figure 2E), but particularly, in HESNs (*r* = 0.71, *p* = 0.0182; Figure 2E). Interestingly, *SLPI* mRNA levels were higher in HESNs than HCs at baseline [0.0268 (*n* = 10) vs. 0.0136 (*n* = 9) mean mRNA RU; *p* = 0.5793], after calcidiol/EtOH [0.0595 (*n* = 14) vs. 0.0085 (*n* = 12) mean mRNA RU; *p* = 0.3260] but only statistically significant after AT2-HIV-1 stimulus regardless calcidiol or EtOH treatments [0.0880 (*n* = 18) vs. 0.0151 (*n* = 26) mean mRNA RU; *p* = 0.0497] (Figure 2F), although viral stimulus does not significantly modify the *SLPI* mRNA expression (Figure 2G).

**Figure 3:**
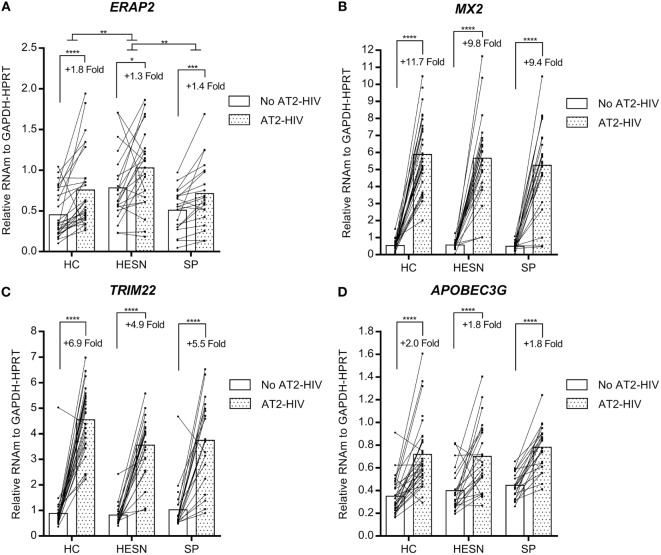
**Viral stimulus increase expression of the aminopeptidase *ERAP2* and antiviral genes in all studied groups**. Calcidiol- and EtOH-treated peripheral blood mononuclear cells from HESNs, HCs, or seropositives (SPs) were combined to analyze differences in the mRNA levels of *ERAP2* (*n* = 20–28) **(A)**, *MX2* (*n* = 21–29) **(B)**, *TRIM22* (*n* = 21–28) **(C)**, and *APOBEC3G* (*n* = 21–29) **(D)** between AT2-HIV-stimulated (dotted bars) and -unstimulated cells (white bars) by paired *t*-test or Wilcoxon test in the case of *TRIM22*. Furthermore, differences between HESN and HCs or SPs in the mRNA levels of these genes were analyzed by ANOVA with multiple comparisons *post hoc*. Bars correspond to means. The viral-induced fold induction is shown. *p < 0.05, **p < 0.01, ***p < 0.001, and ****p < 0.0001 are presented.

In contrast, the mRNA levels of genes encoding the aminopeptidase *ERAP2* and antivirals *MX2, TRIM22*, and *APOBEC3G* were similar between HESNs, HCs, and SPs at baseline and they were not modified by calcidiol treatment (Table S1 in Supplementary Material). However, the AT2-HIV-1 stimulus increases significantly the mRNAs of *ERAP2* (1.8-fold, *p* < 0.0001 in HCs; 1.3-fold, *p* = 0.0178 in HESNs; 1.4-fold, *p* = 0.0004 in SPs); of *MX2* (11.7-fold, *p* < 0.0001 in HCs; 9.8-fold, *p* < 0.0001 in HESNs; 9.4-fold, *p* < 0.0001 in SPs); of *TRIM22* (6.9-fold, *p* < 0.0001 in HCs; 4.9-fold, *p* < 0.0001 in HESNs; 5.5-fold, *p* < 0.0001 in SPs); and of *APOBEC3G* (2.0-fold, *p* < 0.0001 in HCs; 1.8-fold, *p* < 0.0001 in HESNs; 1.8-fold, *p* < 0.0001 in SPs) (Figure 3). Furthermore, *ERAP2* mRNA levels were significantly higher in HESNs than HCs (*p* = 0.0010) and SPs (*p* = 0.0099) after both calcidiol/EtOH and AT2-HIV-1 stimuli (Figure 3A).

### Calcidiol Stimulus Increases the mRNA Levels of *HAVCR2* (TIM-3)

**Figure 4:**
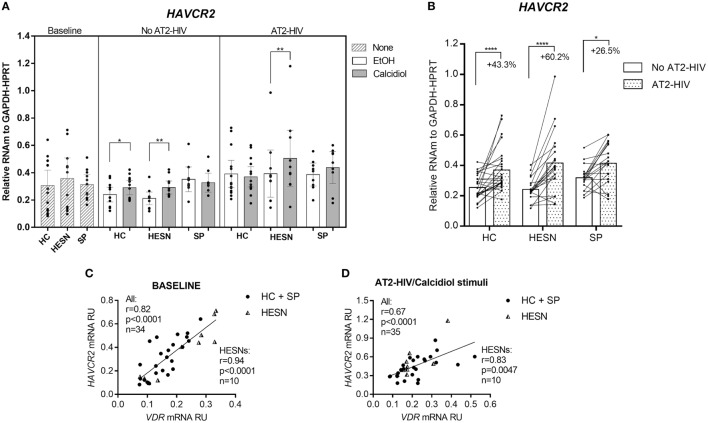
**Calcidiol increases the mRNA levels of *HAVCR2***. Aligned dot plots show the mRNA levels of *HAVCR2* (*n* = 9–14) **(A)**, relative to the glyceraldehyde-3-phosphate dehydrogenase/hypoxanthine-guanine phosphoribosyltransferase mRNA expression in peripheral blood mononuclear cells (PBMCs) from HIV-exposed seronegative individuals (HESNs), HCs, or SPs unstimulated (baseline, striped bars) or cultured with calcidiol 100 ng/mL (gray bars) or EtOH 0.01% v/v (white bars) with or without AT2-HIV-1. Differences in mRNA expression between HESNs and HCs or vs. SPs in PBMCs at baseline were analyzed by one-way ANOVA. Differences between HESNs and HCs or vs. SPs as well as between calcidiol and EtOH treatments in mRNA expression in PBMCs stimulated with or without AT2-HIV-1 were analyzed by two-way ANOVA. Calcidiol and EtOH treatments were combined to analyze differences in the mRNA levels of *HAVCR2* (*n* = 19–26) **(B)** between AT2-HIV-stimulated (dotted bars) and -unstimulated cells (white bars) by paired *t*-test. Correlations of *VDR* mRNA with mRNAs of *HAVCR2* in PBMCs at baseline **(C)**, and of *HAVCR2* in PBMCs under AT2-HIV/Calcidiol stimuli **(D)**, were evaluated using the Spearman coefficient rank, *r*. Bars correspond to means with CI 95%. *p < 0.05, **p < 0.01, and ****p < 0.0001 are presented.

Whereas *HAVCR2* mRNA levels were not different between the groups studied at baseline, with or without AT2-HIV-1 stimuli, incubation with calcidiol increased the *HAVCR2* mRNA levels by 40.6 and 23.9% without AT2-HIV-1 stimulus in HESNs (*p* = 0.0015) and HCs (*p* = 0.0420), respectively (Figure 4A). Likewise, calcidiol increased the *HAVCR2* mRNA levels by 29.4% with AT2-HIV-1 stimulus only in HESNs (*p* = 0.0080) (Figure 4A).

The AT2-HIV-1 stimulus also significantly increase the *HAVCR2* mRNA levels by 43.3% in HCs (*p* < 0.0001), by 60.2% in HESNs (*p* < 0.0001), and by 26.5% in SPs (*p* = 0.0103) (Figure 4B).

Additionally, there were positive correlations between the mRNA of *HAVCR2* and *VDR* at baseline conditions (*r* = 0.82, *p* < 0.0001 in all individuals and *r* = 0.94, *p* < 0.0001 in HESNs; Figure 4C) and after AT2-HIV-1 stimulus (*r* = 0.67, *p* < 0.0001 in all individuals and *r* = 0.83, *p* = 0.0047 in HESNs; Figure 4D).

### Calcitriol Stimulus Increases the Percentage of TIM-3-Expressing CD4+ T Cells

Since we found a calcidiol-induced increase in the *HAVCR2* (TIM-3) mRNA, we also explored if this effect would be maintained at the protein level by evaluating the TIM-3 expression on T and NK cells of healthy controls by flow cytometry after both calcidiol (precursor of active VitD) and calcitriol (active VitD) treatments at physiological concentrations.

**Figure 5:**
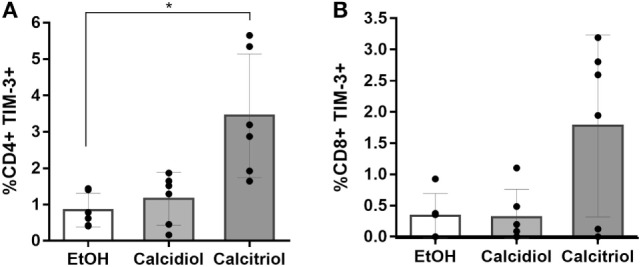
**Calcitriol increases the percentage of TIM-3-expressing CD4+ T cells**. Peripheral blood mononuclear cells from six healthy donors were treated with calcidiol at 250 nM, calcitriol (light gray bars) at 0.5 nM (dark gray bars), or with 0.01% v/v EtOH (white bars) as vehicle control for 48 h and the percentage of CD4+ **(A)** and CD8+ **(B)** T cells-expressing TIM-3 were assessed by flow cytometry. Differences were compared by One-way ANOVA with post hoc Bonferroni’s multiple comparisons. Bars correspond to means with CI 95%. *p < 0.05 is presented.

While in NK cells, any of the VitD treatments did not significantly modify the TIM-3 expression (data not shown), the active (calcitriol), but not the precursor VitD (calcidiol), did significantly increase the percentage of CD4+ T cells-expressing TIM-3 protein compared to EtOH controls (4.14-fold, *p* = 0.0379; Figure 5).

## Discussion

The natural resistance to HIV-1 infection exhibited by HESNs seems to be a genetically determined multifactorial phenomenon involving strong but well-controlled immune responses against HIV-1 possibly triggered by a frequent viral exposure (1–3).

Interestingly, several antiviral factors and effector mechanisms associated with resistance have been shown to be influenced by the VitD pathway (19, 20). Remarkably, similar to previous results in PBMCs and genital mucosa of HESNs from a Colombian cohort (20), higher mRNA levels of the *VDR* were observed in unstimulated PBMCs from HESNs compared to HCs; moreover, the calcidiol-to-calcitriol-activating enzyme *CYP27B1* was also higher in HESNs (Figures 1A,C). These results support the participation of VitD pathway in the natural resistance to HIV-1 infection and suggest an improved ability of HESNs to respond to VitD stimulus.

Yet, the effect of estrogens to the VDR expression could have slightly influenced our results due to the higher proportions of women in HESNs compared to HCs.

Even though in this study we explored the broad response in PBMCs, we expect the VDR expression should be higher within each immune cell subset of HESNs since several immune cells subpopulations that have improved functional capacities in HESNs (30) have been also enhanced by VitD (31–33).

Although, we did not measure calcitriol levels directly, HESNs seemed to be more responsive to viral stimulus than HCs increasing *CYP27B1* mRNA levels. This in turns could favor the calcidiol-to-calcitriol conversion, supporting the 45% yet not significant increase in *CYP24A1* mRNA levels in HESNs by the less bioactive calcidiol (34, 35) and its positive correlation with *VDR* mRNA, as previously proposed (17, 19) (Figure 1). The kinetics in *CYP24A1* expression may explain the absence of a significant increase. Indeed, it has been observed that *CYP24A1* expression in T cells peak after 48 h of VitD treatment (21), compared to the 24 h point in time that we analyzed here. Previous studies demonstrated that calcidiol-to-calcitriol conversion results in antiviral activity against hepatitis C virus activity (36), supporting our assumption.

Since the high expression of antiviral factors are part of the resistant phenotype in HESNs, we explored potential differences in the mRNA expression of several antiviral factors between the groups studied (Table S1 in Supplementary Material). Our results suggest that the viral stimulus triggers the expression of antiviral factors known playing a role in resistance to HIV-1 infection such as *ERAP2, MX2, TRIM22*, and *APOBEC3G* (Figure 3), suggesting that the frequent viral exposure in HESNs could induce higher levels of these molecules as particularly observed for APOBEC3G and MX2 in several cohorts of HESNs (5, 9, 37).

However, other antiviral factors such as *CAMP, PI3*, SLPI, and *ERAP2* were significantly higher in HESNs compared to HCs at baseline as previously described in other sexually exposed cohorts (PBMCs and mucosa) (4, 5, 8), which is maintained independently of additional stimuli (Figures 2 and 3A). Although the differences in *SLPI* mRNA were statistically significant only after AT2-HIV-1 stimulus, this is most likely due to higher sample size since viral stimulus does not significantly modify the *SLPI* mRNA expression (Figure 2G). Interestingly, the viral stimulus *in vitro* reduced mRNA expression not only of *VDR* (Figure 1D), but also of the antiviral genes *CAMP* and *PI3* (Figures 2C,D) 24 h after viral challenge, as observed in other antivirals by co-evolved mechanisms (38), suggesting their high expression in HESNs, could be naturally manifested by genetic determination rather than viral-induced at least in PBMCs. Although the expression of these genes could be also genetically induced in other tissues such as anal/genital mucosa or oral compartments, differences in the expression pattern due to the frequent long lasting HIV-1 exposure could not be ruled out.

Despite the fact that the *CAMP* gene has putative VDREs in its promoter (15), calcidiol treatment was not enough to significantly increase its mRNA expression neither before nor after the viral stimulus in any of the groups evaluated. One possibility is that the HIV-1 stimulus alone is insufficient to produce a sizable calcidiol-to-calcitriol conversion, capable of modulating antiviral genes as observed when PHA/IL-2 stimulus was used (19); nevertheless, there was a 40% increase in *CAMP* mRNA along with a significant positive correlation of *CAMP* mRNA levels with those of *VDR* in HESNs (Figure 2C).

On the other hand, whereas no differences in mRNA levels of *HAVCR2* (encoding TIM-3) were found between HESNs, HCs, and SPs either at baseline or after viral stimulus, calcidiol treatment induced a significant increase in their expression mainly in HESNs regardless of the presence of the virus (Figure 4A). The induction of VitD over *HAVCR2* mRNA expression was also supported by the presence of putative VDREs in the genomic region of this gene (Table S1 in Supplementary Material, verified at http://jaspar.genereg.net/cgi-bin/jaspar_db.pl) and by positive correlations between *VDR* and *HAVCR2* mRNA levels (Figures 4C,D).

Moreover, we found that the active (calcitriol) at physiological concentration, but not the precursor VitD (calcidiol), significantly increases the percentage of CD4+ T cells-expressing TIM-3 protein compared to EtOH controls (Figure 5). Since calcidiol need to be first converted into calcitriol before it can induce changes in gene expression could explain why a 48 h calcidiol treatment increases *HAVCR2* mRNA expression but not the protein levels yet, while 48 h treatment of calcitriol, being already active, was enough to observe an increase in the TIM-3 protein levels.

TIM-3 has been identified mainly as a negative regulator of Th1 and Th17 cell responses (10, 11, 39). However, other reports have provided evidence that under acute stimulation, Tim-3 can promote an effector phenotype in CD8+ T cells (40) and NK cells (41).

Similarly, its role during HIV-1 infection is not clear, since TIM-3 has been associated with susceptibility, exhaustion, and progression (42, 43), while other studies have suggested that TIM-3 blocks the release of HIV-1 *in vitro* (12, 13). Since we have observed that VitD has anti-HIV-1 activity *in vitro* (19), the participation of TIM-3 in the VitD-mediated protection could not be ruled out.

Although the well-characterized cohort of HESNs evaluated in this study could compensate the small sample size limitation, further evaluations with a higher sample size, mRNA evaluations at early time points, and with protein quantification are required to confirm these results.

Despite this limitation, our results suggest that HESNs had higher responsiveness to viral and VitD stimuli compared to HCs, by promoting bio-activation of VitD, and a robust but controlled anti-HIV-1 response.

In summary, according to these and our previous results, we can postulate that natural resistance in HESNs is multifactorial acting progressively but also dynamically among several lines of defense [extensively reviewed in Ref. (25, 30)]. Indeed, the first line of defense could be represented by soluble antiviral factors naturally produced in HESNs in higher amounts such as CAMP, PI3, SLPI, and defensins (3–8), which act inhibiting HIV-1 infection directly interacting with virions and through enhancement of specific anti-HIV-1 cellular mechanisms, thus preventing productive infection locally at the transmission site.

A second line of defense may include a cellular component characterized by a higher responsiveness and strong but well-regulated immune responses. These cells could produce higher amounts of other antiviral proteins in response to virus such as those observed in this study: *MX2, TRIM22, APOBEC3G*, and of immunoregulators such as *ERAP2*, further preventing productive infection.

Finally, evidences suggest that adaptive HIV-specific immune responses may also be triggered in HESNs, which contribute with the control of the establishment of systemic infection and dissemination, constituting a third line of defense against HIV-1 infection.

Importantly, VitD could favor these protective responses regulating immune activation in all the lines of defense.

## Ethics Statement

The study was designed and performed according to the Helsinki declaration (1975 revised in 2000) and was approved by the Ethics Committee of the Santa Maria Annunziata Hospital in Florence. Subjects from a cohort of serodiscordant couples exposed to HIV-1 for the past 12 years (it was established in Tuscany in 1997) enrolled at the Santa Maria Annunziata Hospital in Florence were asked for the participation in this study, explaining them venipuncture procedure to obtain peripheral blood representing a low risk for health. If they agree, blood samples were taken. All subjects provided written informed consent to participate in this study.

## Author Contributions

WA-J and MB conceived and designed the study. DT, FV, SC, FM, and MC contributed with population recruitment and sample taking. WA-J and IS performed the experiments. WA-J analyzed the data and drafted the manuscript. MB, MC, and MR assisted with interpretation of the results and manuscript writing. All the authors approved the final version of the manuscript.

## Conflict of Interest Statement

The authors declare that the research was conducted in the absence of any commercial or financial relationships that could be construed as a potential conflict of interest.

## Supplementary Material

Table S1 **Details in genes analyzed**. Genes symbols, primers sequences, the number of samples and the median and interquartile range of mRNA expression of each gene in each condition is detailed. The presence of putative VDREs in genomic regions of each gene verified in http://jaspar.genereg.net/cgi-bin/jaspar_db.pl were also included.

Figure S1 **Flow cytometry strategy**. Sequential gating for the selection of the target cell populations was performed by the following way: first, a gate to exclude doublets was selected by using a forward-scatter height vs. forward-scatter area (FSC-A) plot, followed by the selection of the lymphocyte region by internal complexity in a side-scatter vs. FSC-A gate. For the viability staining, events negative of the fixable viability dye eFluor 506 were selected, whereas the selection of T lymphocytes was done by gating in CD3+ CD56− cells and NK cells were defined as CD3− CD56+ events. From the CD3+ events, gating of CD4 vs. CD8 was then performed for selection of CD4+ and CD8+ T lymphocytes. Finally, fluorescence minus one controls for TIM-3 expression were performed. Representative plots of CD3+ TIM+ cells under ETOH (0.01%), calcidiol (250 nM), and calcitriol (0.5 nM) are shown.
