# Supplementary figures and images for: High Expression of Antiviral and Vitamin D Pathway Genes Are a Natural Characteristic of a Small Cohort of HIV-1-Exposed Seronegative Individuals
Source: Front Immunol. 2017 Feb 13;8:136. doi: 10.3389/fimmu.2017.00136 (PMC5303892; doi:10.3389/fimmu.2017.00136)

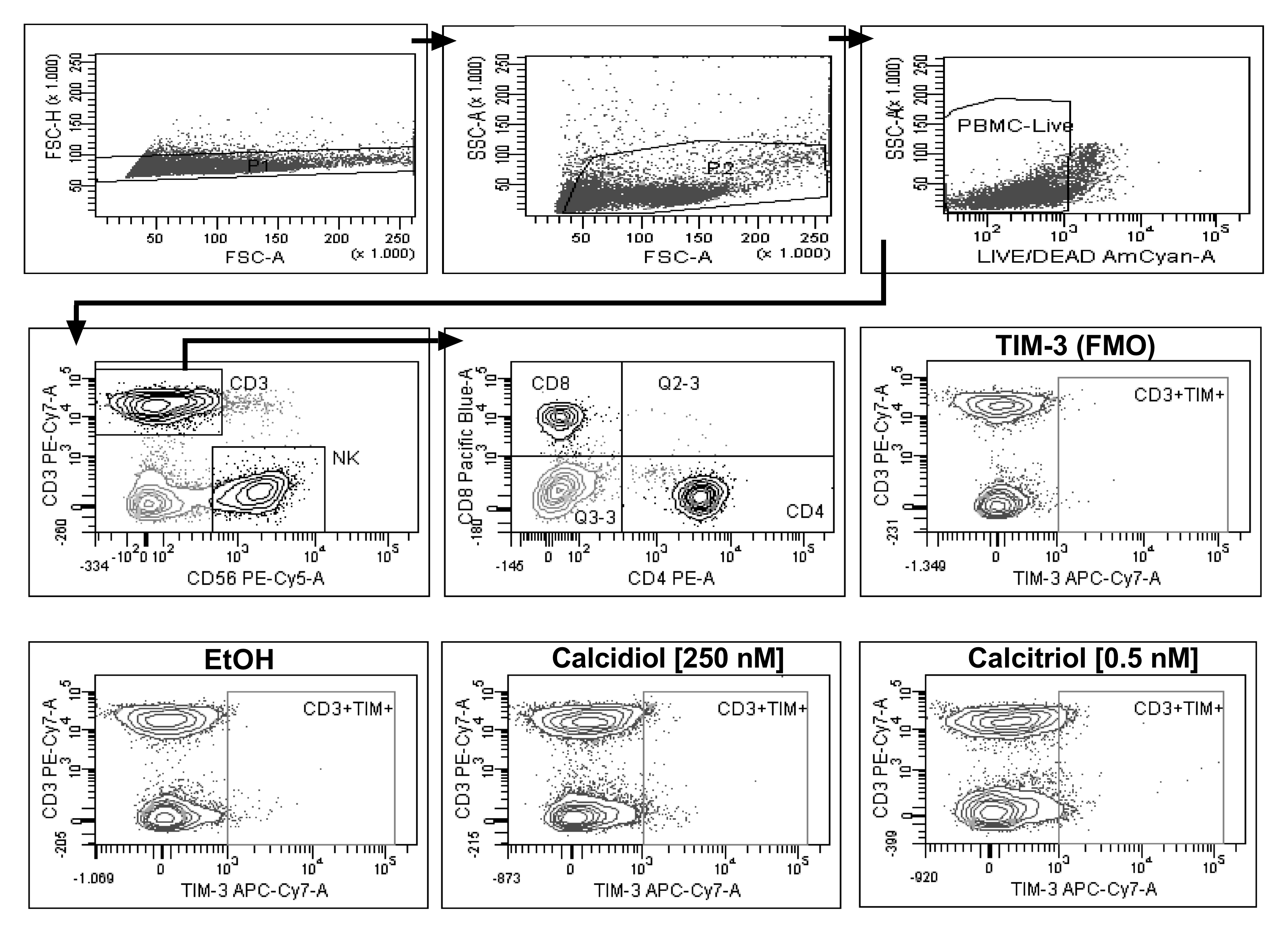

Supplement: Table S1 — Details in genes analyzed. Genes symbols, primers sequences, the number of samples and the median and interquartile range of mRNA expression of each gene in each condition is detailed. The presence of putative VDREs in genomic regions of each gene verified in http://jaspar.genereg.net/cgi-bin/jaspar_db.pl were also included. [file image_1.tif]
